# Discovery of a sugar-based nanoparticle universally existing in boiling herbal water extracts and their immunostimulant effect

**DOI:** 10.1016/j.bbrep.2018.08.004

**Published:** 2018-10-11

**Authors:** Hirofumi Iitsuka, Keiichi Koizumi, Akiko Inujima, Mikiko Suzaki, Yusuke Mizuno, Yoshiki Takeshita, Takeshi Eto, Yoshiki Otsuka, Ryo Shimada, Mengxin Liu, Keisuke Ikeda, Minoru Nakano, Ryo Suzuki, Kazuo Maruyama, Yue Zhou, Hiroaki Sakurai, Naotoshi Shibahara

**Affiliations:** a Division of Kampo Diagnostics, Institute of Natural Medicine, University of Toyama, Toyama 930-0194, Japan; b Graduate School of Medicine and Pharmaceutical Sciences, University of Toyama, Toyama 930-0194, Japan; c Laboratory of Drug Delivery System, Faculty of Pharma-Science, Teikyo University, Tokyo 173-8605, Japan; d Department of Cancer Cell Biology, Graduate School of Medicine and Pharmaceutical Sciences, University of Toyama, Toyama 930-0194, Japan

**Keywords:** SEM, Scanning electron microscope, TEM, Transmission electron microscopy, *G. Radix*, *Glycyrrhizae Radix*, Boiling herbal water extracts, Sugar-based nanoparticle, Immunostimulant effect

## Abstract

Herbal medicine is mainly prepared from boiling herbal water extracts. Many epoch-making immunosuppressant drugs, such as glycyrrhizic acid (old example) and FTY720 (current example), were developed from herbal secondary metabolites in the boiling water extract by partition with organic solvents. However, few immunostimulants have been discovered by this method. Instead of the usual method, we aimed to find a novel immunostimulant component by two unique methods in the research of herbal medicine: ultracentrifugation and electron microscopy. The immunostimulant was not a secondary metabolite, as expected, but the structure was a nanoparticle formed by a polysaccharide. In addition, we clarified the immune effect of the nanoparticle. Intake of the nanoparticle by phagocytosis resulted in immunostimulant effects by increasing the genes and proteins of inflammatory cytokines in macrophage cells. The immunostimulant effects were inhibited by a phagocytosis inhibitor, cytochalasin D. To the best of our knowledge, this study is the first to describe the discovery of a nanoparticle in boiling herbal water extracts and its immunostimulant properties. This study will provide additional understanding of the efficacy of herbal medicine, in that the immunostimulant nanoparticle universally exists in boiling herbal water extracts. Thus, traditional herbal medicine may be an oldest known nanomedicine. Furthermore, this study suggests that the immunostimulant nanoparticle simply can be obtained from herbal medicine only by ultracentrifugation. We hope that this simple strategy will substantially contribute to drug development, including vaccine adjuvant, in the future.

## Highlights

• Nanoparticles are universally present in boiling herbal water extracts.
• Nanoparticles are obtained from these extracts by ultracentrifugation.
• Nanoparticles are mainly composed of polysaccharides.
• Nanoparticles have an immunostimulatory effect on macrophage cells.
• This effect is exhibited by the phagocytosis of exogenous nanoparticles.

## 1 Introduction

In traditional herbal medicine practiced worldwide (Chinese herbal medicine in China, Kampo medicine in Japan, Ayurveda in India, Unani in India, Central Asia, and North African countries, and so on) [1], the medicines are mainly prepared from boiling herbal water extracts [2]. The active components of these herbal medicines have been studied mainly in the form of secondary metabolites. Secondary metabolite products are usually obtained by partition with organic solvents [3]. As a result, a number of excellent modern medical drugs, particularly immunosuppressant drugs, are being developed from the secondary metabolites of these herbal medicines. As a current example, FTY 720 (fingolimod hydrochloride) is a novel immunosuppressant that is a functional antagonist of the first discovered S1P1 receptor, which was synthesized by structural transformation of the secondary metabolite of *Isaria sinclairii* parasitic on *Cordyceps*. FTY 720 is currently used as a drug for treating multiple sclerosis. Studies on secondary metabolites of herbal medicines are yielding tremendous benefits to humanity [4]. Herbal medicines are expected to be attractive candidates for components of new drugs [3].

There have been very few studies on the immunostimulant components of herbal medicines. We suggest that this is because it is hard to find the immunostimulant components in boiling herbal water extracts by partition with organic solvents because these components are not secondary metabolites.

Using two unique methods in the research on herbal medicine, ultracentrifugation and electron microscopy, we discovered a novel unknown nanoparticle in herbal boiling water extract that had an immunostimulant effect. Here we report the discovery of the immunostimulant nanoparticle and describe its properties.

## 2 Materials and methods

### 2.1 Reagents and cells

Lipopolysaccharide (LPS; derived from *Escherichia coli* serotype 0111: B4, no. L-2630) and cytochalasin D were purchased from Sigma–Aldrich Japan (Tokyo, Japan). DiO, DAPI.RAW 264.7 cells (American Type Culture Collection, VA, USA), a murine macrophage cell line, were cultured in DEME medium (Thermo Fisher Scientific) supplemented with 10% (v/v) fetal bovine serum and 1% penicillin and streptomycin at 37 °C in a humidified atmosphere containing 5% CO_2_. All assays were performed when these cells covered 70% of the dish surface.

### 2.2 Isolation of the nanoparticle from boiling herbal water extracts

*Glycyrrhizae Radix, Cinnamomi Cortex, Puerariae Radix*, *Zingiberis Rhizoma, Paeoniae Radix, and Astragali Radix* were purchased from Tochimoto Tenkaido, Osaka, Japan. The boiling herbal water extracts was prepared by boiling a mixture (100 g) of the above herbal medicines gently in 500 mL of water for 50 min and then filtering the decoction. The herbal boiling water extract was centrifuged at 3000×*g* for 5 min (KUBOTA 6800; KUBOTA, Tokyo, Japan), and the supernatant was collected and centrifuged at 20,000×*g* for 20 min. The supernatant was collected and ultracentrifuged at 140,000×*g* for 50 min twice, according to the preparation methods of exosome [5]. After removal of the supernatant, the clear transparent pellet was dispersed in distilled water and freeze-dried.

### 2.3 Transmission electron microscopy (TEM) and dynamic light scattering analysis

The nanoparticles were examined with a JEM-2000EX operated at 100 kV (JEOL Datum, Tokyo, Japan) at the Hanaichi UltraStructure Research Institute, Aichi, Japan. For negative staining with 2% (w/v) uranyl acetate (Cerac, USA), a 400-mesh grid with a carbon support film (10–20 nm in thickness) was used. The sizes of the suspended nanoparticles were confirmed by dynamic light scattering analysis using an FPAR-1000 particle analyzer (Otsuka Electronics, Osaka, Japan).

### 2.4 Elementary analysis and Raman spectroscopy

Elementary analysis was performed as described previously [6]. Elements of the freeze-dried nanoparticle were detected by Electron Probe Microanalyzer, EPMA (JEOL, Tokyo, Japan). Scanning electron microscope (SEM) observations were performed on the nanoparticles (Hitachi High-Technologies, TM3030, Tokyo, Japan). Raman spectroscopy was performed against the powder segment of the freeze-dried nanoparticles following the conditions of inVia Reflex Raman microscopy (Renishaw K.K, Tokyo, Japan), as follows: Laser: LD pumped green laser (532 nm), objective lens magnification×50, irradiating laser beam diameter about 1.5 µm, irradiation laser power 1 mW or less, photometric Raman shift range 4000–150 cm^−1^, wave number resolution about 6 cm^−1^, number of integrations 10, data processing library search by spectral waveform comparison with database (Raman Library “RAMANDB,” JEOL, Tokyo, Japan).

### 2.5 Analysis of sugar content

A sample of 0.3 g of the freeze-dried nanoparticles was added to 3 mL of 72% sulfuric acid at 30 °C for 1 h. This reaction solution was completely transferred to a pressure bottle while being mixed with 84 mL of purified water and then was thermally decomposed in an autoclave at 120 °C for 1 h. After thermal decomposition, the decomposed solution and the residue were separated by filtration. Quantitative analysis of monosaccharides in the decomposed solution was carried out by high-performance liquid chromatography (HPLC) (GL-7400 HPLC system, GL Science, Tokyo, Japan).

### 2.6 Confocal laser fluorescence microscope

The suspension of the nanoparticles was mixed with 3,3′-dioctadecyloxacarbocyanine perchlorate (DiO; final concentration 0.001 mM) (Thermo Fisher Scientific, MA, US). To remove free DiO, the nanoparticles were precipitated with ultracentrifugation (139,000×*g*, 70 min). The sediment containing the nanoparticles labeled with DiO was suspended with phosphate-buffered saline (PBS)(−). The DiO-labeled nanoparticles were added to the RAW 264.7 medium in the glass base dish, incubated for 2 h at 37 °C, and fixed with 4% paraformaldehyde for 10 min. After PBS washing, coverslips were inverted onto a slide with SlowFade Gold Antifade Reagent with DAPI (Life Technologies, CA, US). Finally, the cells were observed with a confocal laser scanning microscope (TCS-SP5, Leica Microsystems, Wetzlar, Germany).

### 2.7 Stimulation of RAW 264.7 cells by the nanoparticles in the presence of cytochalasin D

RAW 264.7 cells (5 × 10^5^) cultured on 12-well plates were cultured for 24 h, and preincubated in the absence or presence of cytochalasin D (50 μmol/L) for 1 h at 37 °C. After preincubation, the nanoparticles (10 μg/mL) were incubated for 6 h and treated. The IL-6 levels in cell culture medium of these cells were determined with a mouse IL-6 ELISA kit (eBioscience, San Diego, CA, USA) according to the manufacturer's instructions. Total RNA in the RAW 264.7 cells was extracted with a RNeasy Mini Kit (Qiagen, Valencia, CA, USA). The cDNAs were amplified with FastStart Essential DNA Green Master (Roche, Pleasanton, CA, USA).

The forward/reverse transcription polymerase chain reaction (RT-PCR) primer pairs for mouse cDNAs were as follows: IL-6 (5′-GCTACCAAACTGGATATAATCAGGA-3′/5′-CCAGGTAGCTATGGTACTCCAGAA-3′); and β-actin (5′-CTAAGGCCAACCGTGAAAAG-3′/5′-ACCAGAGGCATACAGGGACA-3′). Quantitative RT-PCR (qRT-PCR) was performed was performed as described previously [7].

### 2.8 Statistical analysis

Statistically significant differences within each set of categorical data were determined by Tukey–Kramer HSD tests. Statistical analyzes were performed with JMP Pro software version 13 (SAS Institute Japan, Tokyo, Japan). P < 0.05 was considered to indicate statistical significance.

## 3 Results

### 3.1 Discovery and isolation of novel nanoparticles in herbal boiling water extract

**Fig. 1:**
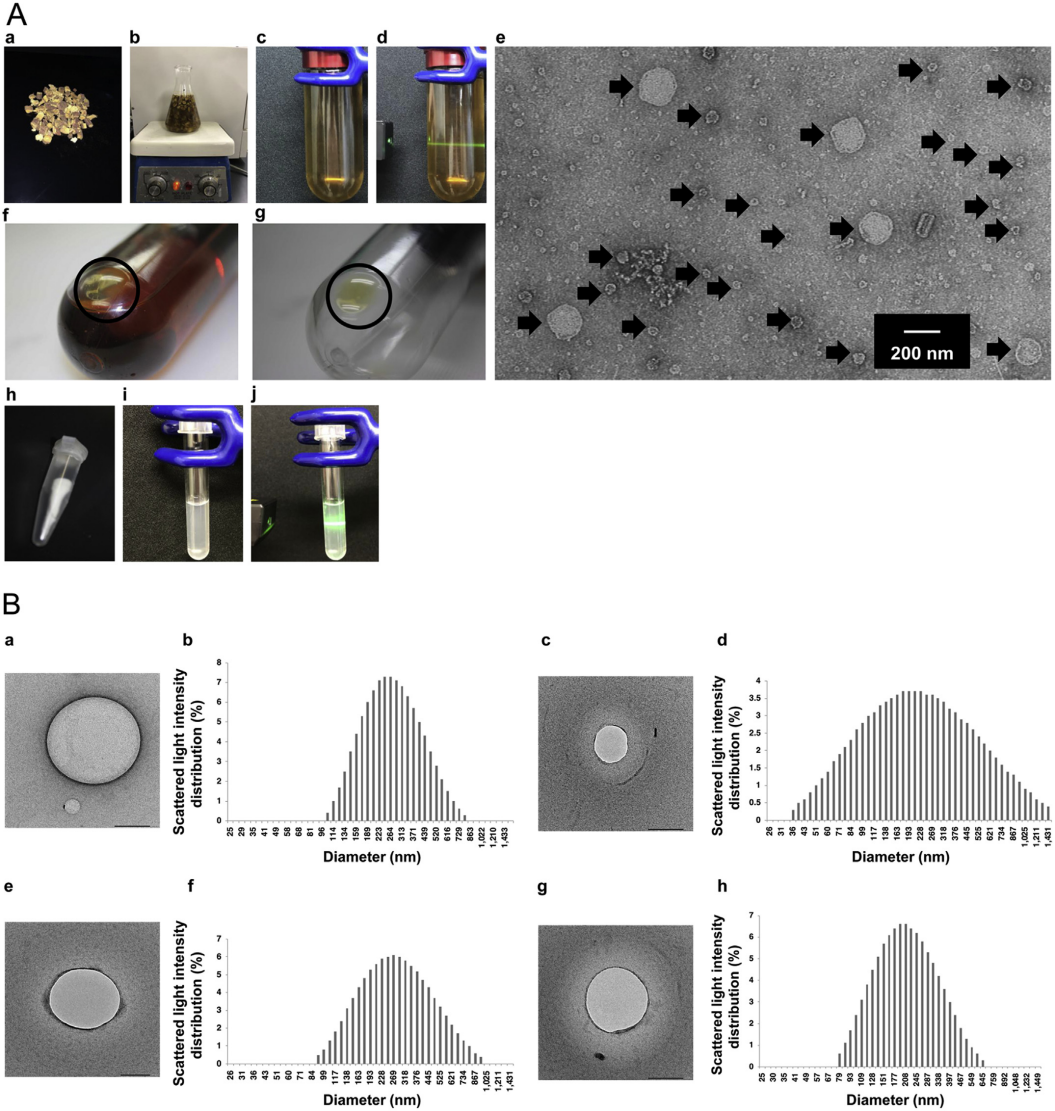
**Novel nanoparticles in boiling herbal water extracts.** (A) Discovery and isolation of the nanoparticles. a: Cut *Glycyrrhizae Radix.* b: boiling *G. Radix* water extract. c: 10 times diluted boiling *G. Radix* water extract after low-speed centrifugation for removal of the residue. d: Irradiation of the extract by laser beam to confirm the Tyndall phenomenon. e: Observation of the nanoparticles (arrows) in the extract by transmission electron microscope (TEM). f and g: After ultracentrifugation, the gel-like precipitate of the nanoparticles (circle) was observed on the bottom. h: Freeze-dried product of the nanoparticle precipitate. i: Good aqueous dispersion of the freeze-dried nanoparticles. The particles did not precipitate without ultracentrifugation. j: The Tyndall phenomenon was also observed in the dispersion, the same as boiling *G. Radix* water extract (d). (B) TEM photographs of the aqueous dispersion of the nanoparticle boiling *G. Radix* water extract (scale bar, 200 nm) (a) and its diameter with a log-normal distribution (b). *Cinnamomi Cortex* (c and d), *Puerariae Radix* (e and f), *Zingiberis Rhizoma* (g and h).

**Fig. S1:**
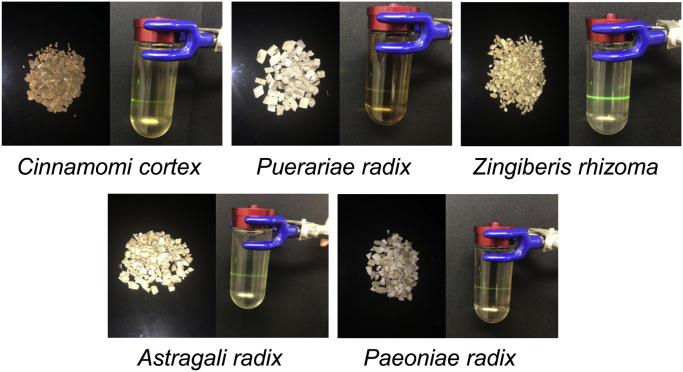
Cut herbal and Tyndall phenomenon in 10 times diluted boiling herbal water extracts, Cinnamomi Cortex (a), Puerariae Radix (b), Zingiberis Rhizoma (c), Astragali Radix (d) and Paeoniae Radix (e).

Initially, we carefully observed various boiling herbal water extracts with naked eye. We were fortunately able to intuitively observe the presence of colloids in these extracts. Subsequently, we attempted to detect the presence of colloids. Because of limitations of space, the extract of *G. Radix* is shown in Fig. 1A as an example of one of the various boiling herbal water extracts. The boiling *G. Radix* water extracts were irradiated by the laser beam (Fig. 1Aa–d, Supplemental Fig. 1). As a result of the Tyndall phenomenon [8], the presence of colloidal particles was tentatively suggested (Fig. 1Ad). The phenomenon was observed in other herbal boiling water extracts (Supplemental Fig. 1). When the extract was observed by TEM, many spherical nanoparticles were observed in all boiling herbal water extracts that were examined in this study (Fig. 1Ae). We next attempted to isolate the nanoparticles from the extract using exosome isolation methods [5]. Using ultracentrifugation, the gel-like nanoparticle precipitate was isolated on the bottom of the tube (Fig. 1Af and g). Fig. 1Bh shows the freeze-dried nanoparticle powder, which has good stability in aqueous dispersion (approximately 50 mg/mL) (Fig. 1Ai). The Tyndall phenomenon was observed in the dispersion, as with the boiling herbal water extracts (Fig. 1Aj).

### 3.2 Morphological features of the nanoparticles

We next observed the dispersed nanoparticles by TEM. The morphological features did not change; the spherical shape was maintained in comparison with that of the nanoparticles in the freeze-dried powder dispersion and *G.Radix* water extract (Fig. 1Ba). The sizes of the nanoparticles of *G. Radix* have a log-normal distribution, with diameters of approximately 100–800 nm (Fig. 1Bb). Similar morphological features, size distributions, and diameters were observed in nanoparticles in other boiling herbal water extracts (Fig. 1Bc–h). These data indicate that nanoparticles are universally present in boiling herbal water extracts.

### 3.3 Analysis of composition of the nanoparticles

**Fig. 2:**
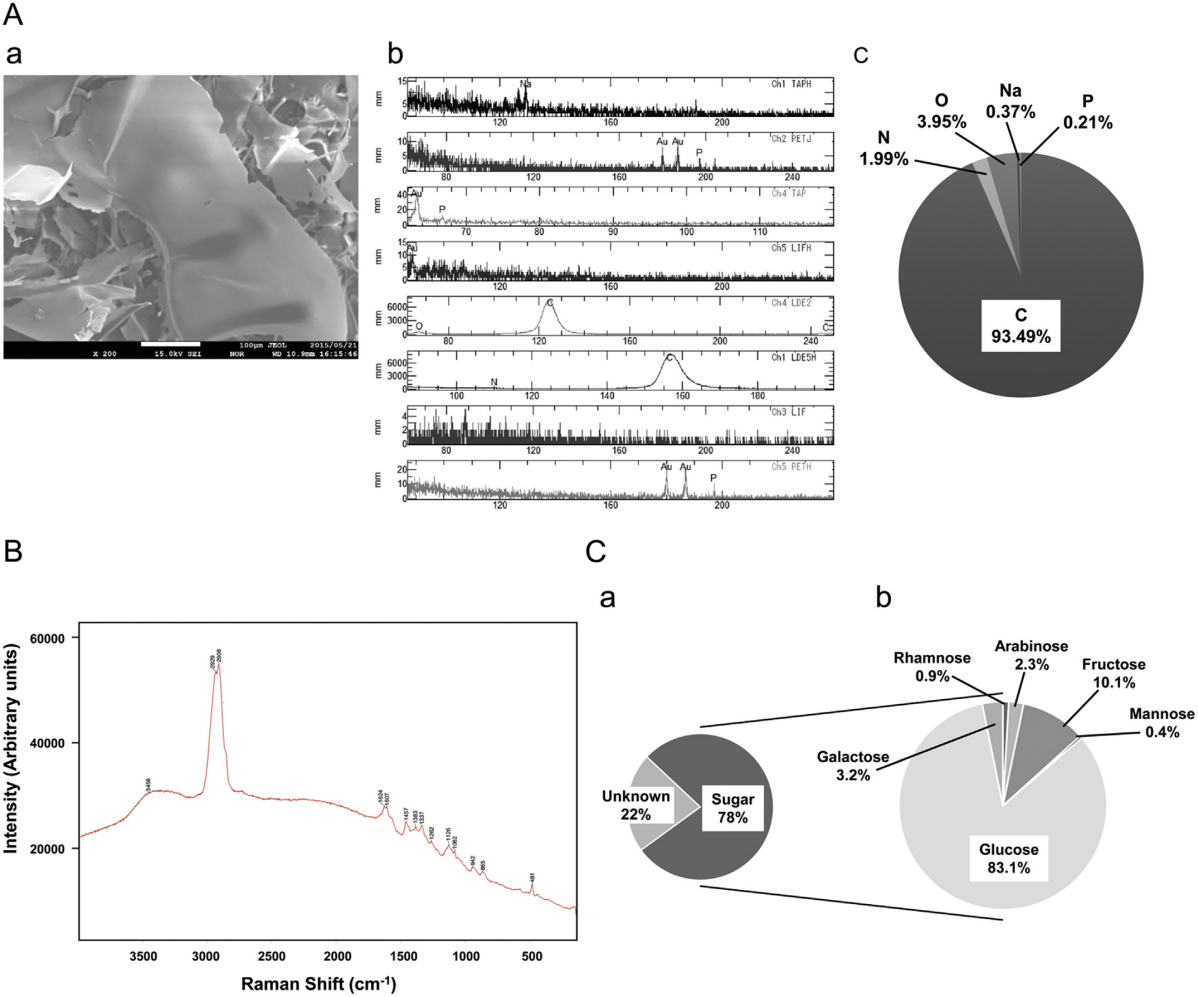
**Analysis of composition of the nanoparticles.** (A) Elemental analysis of the freeze-dried nanoparticles isolated from boiling *G. Radix* water extract by Electron Probe Microanalyzer (EPMA). Representative results of scanning electron microscope (SEM) photographs (scale bar, 100 µm) (a) and EPMA spectra (b) of the nanoparticles. Elemental composition (%) in total weight of the freeze-dried nanoparticles (c). (B) Raman shift spectra (b) of the freeze-dried nanoparticles isolated from boiling *G. Radix* water extract. (C) Composition (%) in total weight of the sugar and the unknown components other than sugar constituting the freeze-dried nanoparticles isolated from boiling *G.Radix* water extract by LC–MS (a). Composition (%) of various monosaccharides in the freeze-dried nanoparticles isolated from boiling *G. Radix* water extract by LC–MS (b).

We next focused on the nanoparticles isolated from boiling *G. Radix* water extracts, because *G.Radix* is a frequently used herb [9]. EPMA analysis (Fig. 2Aa–c) and Raman spectroscopy (Fig. 2B) were performed in order to analyze the composition of the freeze-dried nanoparticles isolated from the boiling *G. Radix* water extracts. As shown in Fig. 2A, the nanoparticles were composed mainly of carbon (93.49%), followed by oxygen (3.95%). In comparison with the similarity of the Raman spectra between the waveform of the freeze-dried nanoparticles and that equipped with the database (Fig. 2B), it was predicted that the nanoparticles of the boiling *G. Radix* water extracts were composed of polysaccharides such as arabinogalactan and cellulose (Table 1). Raman spectra patterns similar to those of *G. Radix* nanoparticles were confirmed in the nanoparticles in other boiling herbal water extracts (data not shown). Various monosaccharides, including, in descending order, glucose, galactose, and arabinose, were detected after sulfuric acid dissolution of the nanoparticles by LC–MS (Fig. 2C). These results suggest that the nanoparticles in boiling herbal water extracts are mainly composed of polysaccharides.

**Table 1:** Library search by spectra waveform comparison with database.

**Number**	**Quality index**	**Spectra waveform identification**
1	0.40	Arabinogalactan
2	0.40	Dry yeast
3	0.45	Diethylaminoethyl cellulose
4	0.50	Cellulose film
5	0.50	Cellulose (clean paper)

### 3.4 Immunostimulant effect of the nanoparticles on macrophages

**Fig. 3:**
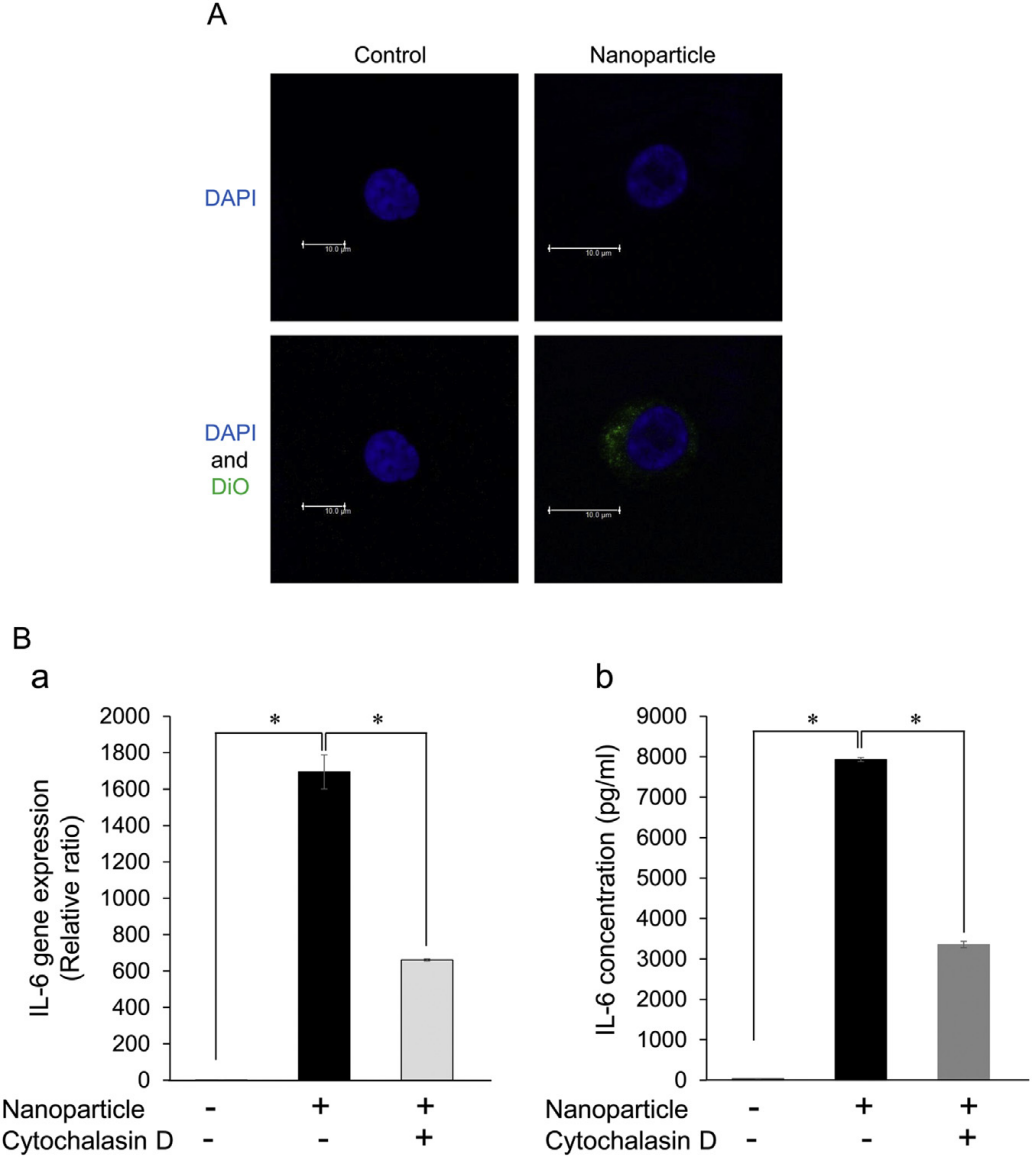
**Immunostimulant effect of the nanoparticles.** (A) Intake of the nanoparticles isolated from boiling *G. Radix* water extract. RAW 264.7 cells were incubated with DiO (green)-labeled nanoparticles or control (PBS (−)) for 1 h at 37 ℃. After fixation, the cells were observed with a confocal laser scanning microscope (scale bar, 10 µm). The nucleus was stained in blue by DAPI. (B) Upregulation of interleukin 6 (IL-6) gene and protein via phagocytosis of the nanoparticles. Expression of the IL-6 gene in RAW 264.7 cells cultured for 24 h in the absence or presence of the nanoparticles was analyzed by quantitative reverse transcription polymerase chain reaction (qRT-PCR) (a) and enzyme-linked immunoassay (ELISA) (b). Expression levels of IL-6 in RAW 264.7 cells using the phagocytosis inhibitor cytochalasin D for control (PBS(−)). *P < 0.01.

Nanoscale materials are easily taken into macrophages by phagocytosis [10]. Therefore, fluorescently labeled nanoparticles isolated from boiling *G. Radix* water extracts were applied to RAW264.7 mouse macrophage cells in vitro [11]. The accumulation of fluorescence showed that the nanoparticles were taken into the macrophages (Fig. 3A). We also confirmed the upregulation of the inflammatory cytokine gene *IL-6* in RAW 264.7 cells by qRT-PCR (Fig. 3Ba) [12] and the upregulation of the inflammatory cytokine IL-6 protein in RAW 264.7 cells by ELISA (Fig. 3Bb). Similar upregulations were confirmed in nanoparticles isolated from other herbal medicine water extracts (data not shown). The upregulations were blocked by the actin polymerization inhibitor cytochalasin D as a result of inhibition of the phagocytosis of the nanoparticles (Fig. 3B) [13]. However, in a small interfering RNA (siRNA) experiment, dectin-1 was not a receptor for the nanoparticles (data not shown). These results suggest that the nanoparticles in boiling herbal water extracts have bioactivity, in particular an immunostimulant effect, via phagocytosis of the exogenous nanoparticles.

## 4 Discussion

As mentioned in the Introduction, the mechanism of the pharmacological efficacy of herbal medicines has been mainly investigated in secondary metabolites of herbal medicines. For example, glycyrrhizic acid is a secondary metabolite of *G. Radix*, which is the source of the herbal medicines focused on in this study [14]. Glycyrrhizic acid is used worldwide as a component of pharmaceuticals because it has a strong immunosuppressive (anti-inflammatory immunosuppressant) effect [15], [16]. In this way, a number of immunosuppressive components have been identified in secondary metabolite products from herbal medicines, and the molecular mechanism of this immunosuppressive effect has also been elucidated in detail. There are also numerous reports of the immunostimulant effects of herbal medicines from basic research [17] and clinical research [18]. However, immunostimulant components have not yet been discovered in these secondary metabolite products. We previously reported that juzentaihoto, a kampo (Japanese herbal medicine) formula, increased and prolonged antibody production after influenza vaccination in a clinical experiment [19]. We have been studying immunology and nanomedicine for a long time [20], [21], [22], [23]; however, we were not able to identify immunostimulant components from the Japanese herbal medicines, such as juzentaihoto, by partition with organic solvents.

We considered that it was impossible to discover immunostimulant components in herbal medicines by partitioning with organic solvents. Therefore, we used two methods that had not been previously used in research on herbal medicines, electron microscopy and ultracentrifugation of water extracts. Using these methods, we found a novel immunostimulant component in boiling herbal water extracts (Fig. 1, Fig. 2, Fig. 3). These methods may be useful for the discovery of new natural drugs in the future.

The novel immunostimulant component discovered in this study was composed mainly of polysaccharide (Fig. 2). An axis between the polysaccharide β-glucan and dectin-1 has been studied as an immunostimulant component [24]. Dectin-1 is the β-glucan receptor [25]. Increasing β-glucan stimulation by dectin-1 induces the production of inflammatory cytokines, such as tumor necrosis factor α (TNF-α) and interleukin 6 (IL-6), via the signal pathway [26]. However, in a siRNA experiment, dectin-1 siRNA could not decrease upregulation of the activity of NF-κB, which is a central transcription factor in the inflammatory cytokine, by the nanoparticle isolated from boiling *G.Radix* water extracts (data not shown). Thus, these findings suggest that dectin-1 is not the receptor of the nanoparticle isolated from boiling *G. Radix* water extracts.

As expected in this study, the novel immunostimulant component discovered in boiling herbal water extracts was not a secondary metabolite product but was mainly sugar (Fig. 2). On the other hand, unexpectedly but interestingly, the immunostimulant component is a nanoparticle structure formed by the polysaccharide (Fig. 1, Fig. 2). The nanoparticles in all boiling herbal water extracts investigated in this study had diameters of several hundred nanometers. Coincidentally, these diameters are of optimal size for phagocytosis [27]. To determine how immunostimulation occurred, we further investigated phagocytosis (Fig. 3A).

There are many reports that phagocytosis is enhanced by herbal medicine in various cells, including macrophages [28]. Exogenous nanoparticles, such as virus particles and artificial nanoparticles (nanometals, nanominerals, etc.) taken in by phagocytosis are known to enhance immune effects via inflammasomes [29]. As a result, the IL-6 protein and gene expression were elevated by phagocytosis of the nanoparticles isolated from boiling *G. Radix* water extracts (Fig. 3B). This elevated gene expression was inhibited by the phagocytosis inhibitor alone, cytochalasin D (Fig. 3B). Considering that the inhibition was incomplete, the nanoparticles may exert their immunostimulant effect by other unknown molecular mechanisms in addition to phagocytosis. We plan to seek the upstream signal pathways of the inflammatory cytokines controlled by the nanoparticles.

**Fig. 4:**
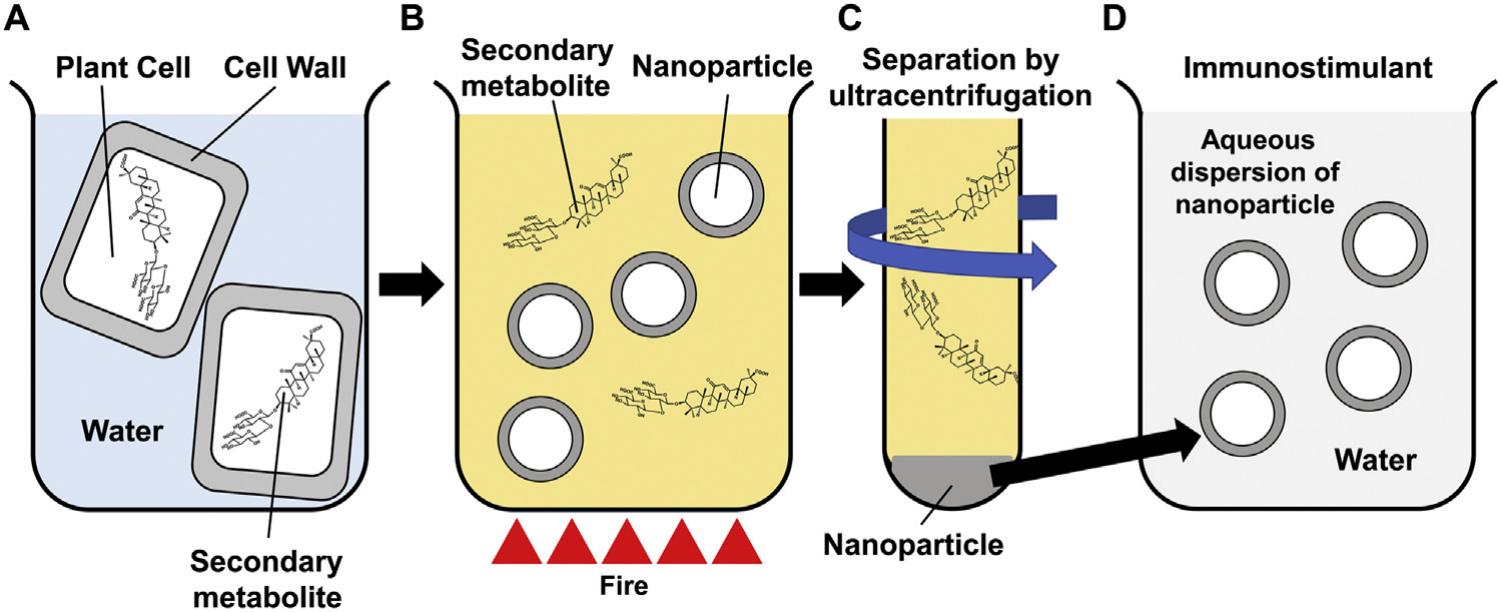
Scheme of the new understanding of the immune efficacy of herbal medicine based on the nanoparticle and its application for drug development. When the plant cells of herbal medicines (A) was boiled in water, their secondary metabolite transferred into water, and the nanoparticle was generated from cell wall degradant (B). The coexistence state of immunostimulant (the nanoparticle) and immunosuppressant (secondary metabolite) in herbal boiling water extract (B). The immunostimulant fraction (D) can simply be separated by ultracentrifugation (C).

We do not know how the nanoparticles are made. However, the two-retrieval keyword, arabinogalactan and cellulose, shown in Raman spectroscopy (Table 1), provided hints to clarify how the nanoparticles were made [30]. Arabinose and galactose (constituents of arabinogalactan) and glucose (constituent of cellulose) were detected in LC–MS (Fig. 2C). Arabinogalactan and cellulose are major components of the cell wall of plants. Therefore, we believe that nanoparticle is a semiartificial assembly form of cell wall degradant produced on boiling (Fig. 4A and B). We also believe that the findings of this study will provide additional understanding, which is based on the immunostimulant nanoparticle, of the pharmacological efficacy of herbal medicines. The boiling herbal water extracts includes immunostimulant nanoparticles and immunosuppressant (secondary metabolites), which possess contrary effects (Fig. 4A and B).

In conclusion, to the best of our knowledge, this is the first study to (1) discover the novel immunostimulant nanoparticle that universally exists in boiling herbal water extracts and (2) obtain nanoparticle by ultracentrifugation, which is a simple method (Fig. 4C). This study highlighted the fact that traditional herbal medicine is an oldest nanomedicine. In the future, we plan to develop a latest nanoparticle adjuvant of vaccine from the oldest nanomedicine, i.e., herbal medicine [17], [18], [19]. We strongly believe that the simple method suggested in this study, which can easily be applied to pharmaceutical manufacturing processes, will result in a great contribution to drug development (Fig. 4D).
